# Missed Diagnosis of Clear Cell Renal Cell Carcinoma on Noncontrast Computed Tomography: The Potential Role of Artificial Intelligence in Reducing Radiological Diagnostic Error

**DOI:** 10.1155/crin/5359978

**Published:** 2026-09-29

**Authors:** Fabio Massimo Abenavoli, Omar Tujjar, Andrea Alfonso

**Affiliations:** ^1^ AVOCA Clinic, Dublin, Ireland; ^2^ Policlinico Di Liegro, Rome, Italy; ^3^ National Orthopaedic Hospital Cappagh, Dublin, Ireland; ^4^ Dept of Anaesthesia, Cambridge University Hospital NHS Foundation Trust, Cambridge, UK, nhs.uk

**Keywords:** anchoring bias, artificial intelligence, Bosniak classification, clear cell renal cell carcinoma, computed tomography, diagnostic error

## Abstract

**Background:**

Clear cell renal cell carcinoma (ccRCC) is the most common histological subtype of malignant renal neoplasms, with an increasing incidence in Western countries. Early diagnosis is a critical determinant of prognosis, as advanced‐stage disease with metastatic dissemination entails a significantly reduced median survival. Characterizing complex cystic renal lesions presents a recognized diagnostic challenge on noncontrast computed tomography (CT), which often leads to underestimation and missed diagnoses. Artificial intelligence (AI) applied to diagnostic imaging is emerging as a supplementary decision‐support tool, but its integration must be critically evaluated to understand its potential to reduce diagnostic errors as well as its limitations.

**Case Presentation:**

We report the case of a 73‐year‐old male physician with a history of myocardial infarction who presented with left flank pain. An initial noncontrast CT of the urinary tract on January 15 revealed a 37‐mm left upper‐pole renal cyst and a 5‐mm suspected calculus. The cyst was characterized as simple, and the report offered only a generic follow‐up recommendation. Over the next 4 months, the patient’s persistent pain was attributed entirely to presumed ureteric stone disease, leading to a ureteroscopy and ureteral stent placement, without characterizing the known renal cyst with contrast. On June 5 (5 months after the initial CT), a contrast‐enhanced CT revealed a 6‐cm heterogeneous, exophytic left renal mass. Subsequent staging confirmed pulmonary and cerebral metastases from ccRCC. The patient died in mid‐August, 7 months after the initial CT. Retrospective re‐evaluation of the baseline CT images, both by a senior radiologist and through an AI platform, confirmed that the lesion already exhibited features suspicious for neoplasia at the time of the first scan, warranting further investigation with contrast‐enhanced CT or magnetic resonance imaging in accordance with international guidelines. Simulated retrospective analysis via a web‐based AI platform not only flagged the mass as suspicious but also generated several critical errors (e.g., hallucinating contrast enhancement on the unenhanced scan, mislocalizing the mass to the mid‐to‐lower pole, and claiming it was “new” without comparison data).

**Conclusions:**

This case demonstrates how a complex cystic renal lesion may be underestimated in the absence of contrast medium and an adequate level of clinical–radiological suspicion. Re‐evaluation by AI produced a result consistent with that of the expert radiologist, suggesting that systematic integration of AI tools into the radiological workflow could contribute to reducing diagnostic errors, particularly in settings where interobserver variability represents a clinically significant risk factor. AI can act as an effective supplementary decision‐support tool to reduce perceptual errors; clinicians and radiologists must remain highly vigilant regarding AI‐generated factual inconsistencies. Proper adherence to imaging guidelines and the use of contrast medium remain paramount.

## 1. Introduction

Clear cell renal cell carcinoma (ccRCC) accounts for approximately 70%–80% of all renal carcinomas and represents the third most common urological malignancy after prostate and bladder cancers [1]. Its global incidence has been rising progressively, in part due to the wider availability of abdominal imaging techniques, leading to an increase in incidental diagnoses of renal masses [2]. The prognosis of ccRCC depends substantially on the stage at diagnosis: the 5‐year survival rate for localized disease exceeds 90%, whereas for metastatic disease, it falls below 15% [3]. Accordingly, the timeliness and accuracy of the initial diagnosis represent determining factors for clinical outcomes.

The characterization of complex cystic renal lesions constitutes a well‐established challenge in radiology. The Bosniak classification is the standard reference system for stratifying the risk of malignancy in renal cysts [4, 5]. This classification typically requires the use of contrast‐enhanced computed tomography (CT) to evaluate wall characteristics, septa, and solid component enhancement. When the examination is conducted without contrast medium, the discriminative capacity between simple and complex cystic lesions is significantly reduced, posing a substantial risk of underestimating lesions that merit further investigation [5].

Artificial intelligence (AI) is finding increasing application in diagnostic imaging, with particular reference to automated image analysis for the detection, segmentation, and classification of lesions [6]. Several studies have demonstrated that systems based on convolutional neural networks can achieve levels of accuracy comparable to, or exceeding, those of radiologists in specific settings [7]. However, clinical adoption remains heterogeneous, and systematic integration into diagnostic workflows is not yet standardized. Furthermore, AI platforms are prone to errors and hallucinations, which are not yet fully understood in real‐world clinical applications.

This case report, drafted in accordance with the CARE (CAse REport) guidelines [8], describes a patient in whom an indeterminate left renal lesion on a baseline noncontrast CT was mischaracterized as a simple cyst. The resulting diagnostic delay—compounded by clinical anchoring bias on ureteric stone disease—was followed by rapid disease progression to metastatic stage and death. Retrospective re‐evaluation by a senior radiologist and by an AI platform confirmed the suspicious nature of the lesion from the first scan. The case is intended to illustrate how interobserver variability in radiological reporting constitutes a concrete clinical risk factor, and how the integration of AI may represent a useful complement to diagnostic practice and to evaluate the retrospective findings of a web‐based AI platform, detailing both its potential to assist radiologists and its notable technical errors.

## 2. Case Presentation

### 2.1. Patient Information

The patient was a 73‐year‐old male physician who presented to a urologist reporting persistent pain in the left flank. The pain was not accompanied by hematuria, fever, weight loss, or other systemic symptoms. His past medical history was significant for a previous myocardial infarction, for which he was receiving antiplatelet therapy with acetylsalicylic acid (aspirin). No other comorbidities of note were reported. The patient had a body mass index (BMI) over 25, was a nonsmoker, did not use alcohol, and had no relevant family history of renal neoplasms or psychosocial factors affecting his care.

### 2.2. Clinical Findings

At the initial consultation, a physical examination revealed pain localized on the left flank, without palpable masses or tenderness on deep palpation. The patient’s general clinical condition was preserved, with no signs of systemic compromise. A preliminary office ultrasound examination performed by the urologist did not reveal any evident renal pathology, apart from a mild dilatation of the renal pelvis.

### 2.3. Timeline of Events

#### 2.3.1. Diagnostic Assessment

##### 2.3.1.1. Baseline Noncontrast CT and Objective Description

The initial CT scan was performed on January 15 using a spiral technique, without intravenous contrast medium and without oral opacification of the bowel loops. Within the limits of this unenhanced examination, the official report described kidneys in normal position and size with apparently normal pyelocalyceal systems. On the left, a hyperdense formation of approximately 5 mm was identified near the pelvi–ureteric junction and attributed to a renal calculus, along with a left upper‐pole renal cyst with a maximum transverse diameter of approximately 37 mm. The report concluded with: “Clinical verification and possible further diagnostic investigation, if clinically deemed necessary, are recommended,” without, however, specifying the need for contrast‐enhanced imaging to characterize the cystic lesion (See Table 1).

**TABLE 1:** Chronological flow of the patient’s diagnostic, clinical, and therapeutic events.

Date	Event and diagnostic procedure	Clinical findings & outcome
January 15	Baseline noncontrast CT scan (urinary tract)	Left upper‐pole renal lesion (37 mm) reported as a “simple cyst.” Left pelvi–ureteric junction calcification (5 mm) interpreted as renal calculus. Generic recommendation for clinical correlation
Late January	Clinical diagnostic interpretation	Treating urologist attributes left flank pain to the 5‐mm calculus. Presumes calculus migrated into the ureter after a negative follow‐up abdominal X‐ray
February 10	Ureteroscopy and ureteral stenting	Attempted ureteroscopy to treat presumed stone; the entire ureter could not be traversed. A ureteral stent is placed for 3 months. Left flank pain persists
June 5 (5 months later)	Diagnostic contrast‐enhanced CT scan	Large exophytic, heterogeneous left renal mass (60 mm) with a thick contrast‐enhancing rim and central necrosis. Bilateral multiple pulmonary nodules and brain metastases identified
June ‐ July	Histopathological diagnosis and clinical decline	CT‐guided biopsy of a lung metastasis confirms metastatic clear cell renal cell carcinoma (ccRCC, Fuhrman Grade 2). Targeted systemic therapy (nivolumab and cabozantinib) scheduled but aborted due to rapid clinical decline. Admission for hematemesis and melena secondary to duodenal metastatic ulceration
Mid‐September (8 months later)	Patient death	Patient experiences progressive multiorgan decline and passes away approximately 8 months after the baseline CT scan

The original CT report stated the following: “Exam performed using spiral technique, without IV contrast injection and without oral opacification of bowel loops for the study of the urinary tract. Within the limits of this baseline‐phase‐only examination, the findings were as follows: the kidneys demonstrated normal position, size, and morphology without excretory dilatation. Right side: parapelvic calcific concretions, not definitively urolithiasic, were attributed to vascular calcifications. Left side: a hyperdense formation (approx. 5 mm) near the pelvi‐ureteric junction consistent with a renal calculus and an upper‐pole renal cyst (approx. 37 mm) were detected. Additional findings included an esophageal hiatal hernia, a distended bladder with wall thickening, and diffuse vascular calcifications. Hepatic, pancreatic, splenic, and adrenal structures were within normal limits. Clinical verification and further diagnostic workup, if deemed necessary, are recommended.

A retrospective, objective analysis of the baseline noncontrast CT images by an expert radiologist revealed that the left upper‐pole mass did not exhibit the classic features of a straightforward simple cyst. The mass was relatively lobulated, and its internal attenuation was not unequivocally fluid or homogeneous.

Furthermore, mild stranding and alteration were noted in the adjacent perirenal fat, which, while nonspecific, further underscored the complex nature of the lesion. According to the joint European Association of Urology (EAU) and American College of Radiology (ACR) guidelines, an indeterminate 37‐mm renal lesion identified on a noncontrast CT must be characterized using contrast‐enhanced imaging to evaluate for solid components, wall thickening, or septations, corresponding to Bosniak Categories IIF, III, or IV. The absence of such further investigation represented the initial point of diagnostic failure.

### 2.4. Contrast‐Enhanced CT and Staging

Following a period of clinical focus on stone disease, a contrast‐enhanced CT was eventually performed on June 5 (5 months after the initial scan). This study revealed a large, exophytic left renal mass affecting the anterior lip of the left kidney, measuring approximately 6 cm in maximum diameter. The mass was heterogeneous, presenting a central necrotic hypodense area surrounded by a thick, irregular rim of strong contrast enhancement, a configuration highly indicative of renal cell carcinoma. Subsequent staging with chest and brain CT documented the presence of pulmonary and cerebral metastatic deposits.

#### 2.4.1. Pathological and Histological Findings

A CT‐guided core needle biopsy of one of the pulmonary metastatic lesions was performed to establish a definitive diagnosis. Histopathological evaluation revealed nested clusters of polygonal cells with abundant, optically clear cytoplasm, uniform nuclei, and a prominent, delicate branching capillary network, characteristic of Fuhrman Grade 2 ccRCC. Immunohistochemical (IHC) profiling demonstrated strong positivity for PAX8, CD10, vimentin, and RCC antigen, while showing negativity for CK7 and CD117, confirming the diagnosis of metastatic ccRCC.

### 2.5. AI Platform Simulation and Discrepancy Analysis

To explore the potential role of AI as a decision‐support tool, the baseline unenhanced CT images were submitted retrospectively to a web‐accessible AI platform (ctread.com, Version 1.2). The platform was provided with only the most significant images provided by the expert radiologist who reviewed the entire CT sequence after complete de‐identification of the DICOM files, removing all direct patient identifiers (including name, date of birth, medical record number, and hospital metadata) to ensure full compliance with HIPAA and GDPR data protection regulations. No clinical history, prior examinations, or targeted prompts were entered. The upload was performed locally.

The AI platform generated an automated diagnostic report within seconds, correctly flagging the left kidney mass as highly suspicious and recommending referral to urology and further contrast‐enhanced imaging (shown in Figure 1).

**FIGURE 1:**
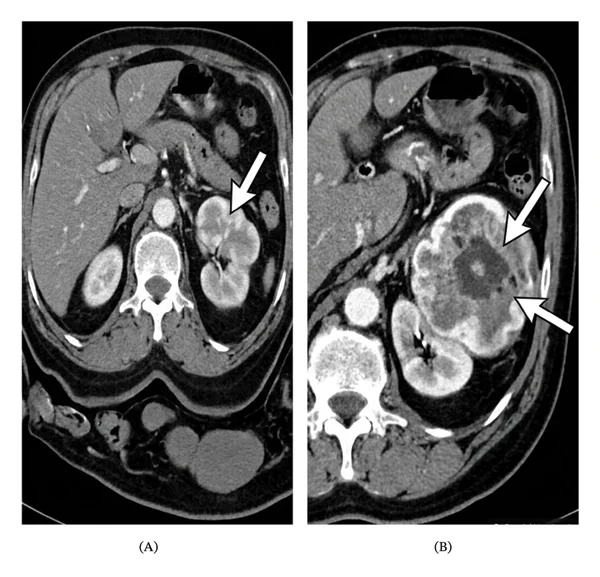
Side‐by‐Side CT imaging comparison. (A) Baseline noncontrast CT scan performed in January, showing a 37‐mm left upper‐pole renal mass (indicated by white arrow), which was initially characterized as a simple fluid cyst but exhibited highly indeterminate characteristics. (B) Follow‐up contrast‐enhanced CT scan performed 4.5 months later in June, demonstrating a 6.0‐cm heterogeneous, exophytic renal mass (indicated by white arrow) showing prominent peripheral contrast enhancement and central necrosis.

**Recommendations:** •Refer to urology for further assessment and possible biopsy of the left renal mass; • consider follow‐up with contrast‐enhanced CT or MRI of the abdomen and pelvis to evaluate for any changes in the lesion or potential metastasis; and •evaluate serum creatinine and electrolytes to assess renal function. **Additional information notes:** “Need an evaluation of the left kidney where there is a new growth.”

However, a detailed analysis of the AI report revealed several major factual errors and technical inconsistencies:

1. Contrast hallucination: The AI report described “heterogeneous enhancement on contrast imaging” and referred to an “enhancing lesion” in the left kidney, despite the uploaded baseline scan being entirely unenhanced (noncontrast). This suggests a significant limitation in the AI’s ability to automatically distinguish unenhanced hyperdense soft tissue from true postcontrast enhancement.
2. Mislocalization: The AI localized the mass to the “mid to lower pole” of the left kidney, whereas both the original radiological report and the subsequent contrast CT confirmed that the lesion originated from the left upper pole.
3. Prior comparison error: The AI report labeled the lesion as “new,” despite no prior examination being uploaded or available to the platform for comparison.

These technical discrepancies demonstrate that while AI can successfully draw attention to a suspicious abnormality (acting as an effective perceptual aid), its written outputs can contain significant factual errors. Radiologists and clinicians must critically review all AI‐generated reports and must not present AI findings as independent clinical confirmation of human interpretations.

### 2.6. Therapeutic Interventions and Clinical Course

In the interval between the first and second CT, the patient’s persistent left flank pain was attributed to the 5‐mm renal calculus, which was no longer visible on subsequent abdominal X‐rays and was presumed to have migrated. A urologist performed a ureteroscopy; however, it proved impossible to traverse the entire length of the ureter due to narrowing. A ureteral stent was placed to remain in situ for 3 months. This clinical focus on stone disease delayed contrast‐enhanced characterization of the known renal mass.

Following the histopathological confirmation of metastatic ccRCC in June, systemic therapy involving nivolumab (an anti‐PD‐1 immunotherapy) and cabozantinib (a tyrosine kinase inhibitor blocking tumor growth and angiogenesis) was scheduled. Unfortunately, due to the patient’s rapid clinical deterioration, this treatment could not be initiated.

The clinical course was characterized by rapid and aggressive disease progression. Approximately 7 months after the baseline CT (mid‐August), the patient was admitted to the hospital with melena. A contrast‐enhanced CT performed on admission documented a marked increase in the left renal mass, which had reached approximately 10 cm in maximum diameter, along with metastatic dissemination to the pancreas, contralateral kidney, stomach, and duodenum. Extensive mucosal ulceration of the gastroduodenal tract was identified as the source of the hemorrhage. Within a few weeks, due to progressive multiorgan decline and the lack of any viable antineoplastic therapy, the patient was transitioned to supportive and palliative care and died in end‐August, exactly 7 months after the initial baseline CT.

## 3. Discussion

### 3.1. Diagnostic Error and the Multistep Pathway Failure

Perceptual and cognitive errors in radiology are well‐documented, with retrospective studies estimating error rates between 3% and 5% in daily clinical practice [9]. In the case of complex cystic renal masses, interobserver variability in the Bosniak classification is a significant challenge, with several studies reporting only moderate agreement (Cohen’s kappa 0.40–0.60), particularly for Intermediate Categories IIF and III [10].

However, this case demonstrates that the diagnostic delay was not merely a single radiological reporting error, but rather a multistep failure pathway involving three distinct stages. First, the initial reporting radiologist undercharacterized the 37‐mm left upper‐pole mass as a simple renal cyst on the baseline unenhanced CT, failing to highlight its indeterminate features. Second, the recommendation in the initial report was formulated in excessively generic and nonspecific terms (“clinical verification and possible further diagnostic investigation, if clinically deemed necessary”), which failed to convey clinical urgency or specify the necessity of contrast‐enhanced CT or MRI as mandated by guidelines [11]. Third, a profound clinical anchoring bias occurred during subsequent urological follow‐up. The treating clinical team focused entirely on the presumed migrating 5‐mm ureteric stone, performing an invasive ureteroscopy and placing a ureteral stent, while leaving the known, indeterminate 37‐mm renal lesion completely uncharacterized for several months despite persistent ipsilateral symptoms.

### 3.2. CT and MRI Characterization of Renal Masses

The characterization of renal masses heavily relies on multiphase contrast‐enhanced CT or MRI. As reviewed by Baytok et al. [12], noncontrast CT alone has major limitations, as simple cysts and complex, necrotic, or solid renal cell carcinomas can appear similar on unenhanced images. Clear cell RCC typically demonstrates strong, heterogeneous enhancement in the corticomedullary phase due to its rich vascularity, along with central areas of low attenuation representing necrosis or cystic changes. On MRI, ccRCC shows high signal intensity on T2‐weighted images and signal loss on opposed‐phase chemical shift imaging due to intracellular lipids, with diffusion restriction reflecting high cellularity. When a lesion on noncontrast CT shows intermediate attenuation (> 149312220 HU), lobulation, or perirenal stranding, it must be considered indeterminate, requiring immediate contrast‐enhanced CT or MRI to exclude malignancy.

### 3.3. The Supplementary Role of AI in Diagnostic Radiology

The AI’s retrospective performance in this case underscores both its promise and its current limitations. While the AI successfully flagged the lesion as suspicious—aligning with the expert radiologist’s retrospective assessment—the presence of severe factual errors in its output (hallucinating contrast enhancement on an unenhanced scan, mislocalizing the lesion, and declaring it “new”) shows that AI cannot yet be trusted as an independent diagnostic reader. Rather, AI should be utilized strictly as a supplementary decision‐support tool (CAD) to reduce perceptual errors, particularly in high‐workload environments or where double‐reading is unavailable.

### 3.4. Strengths and Limitations

The primary strength of this case report is the comprehensive documentation of the patient’s entire clinical trajectory, reconciling the previously inconsistent timelines. The main limitation is that this is a single retrospective case study, which carries inherent hindsight bias. The AI platform used (ctread.com) has not undergone independent clinical validation in peer‐reviewed literature, and its factual inconsistencies demonstrate the need for rigorous, large‐scale prospective clinical trials before clinical adoption.

## 4. Conclusion

In conclusion, this case illustrates that diagnostic delay in ccRCC is often a multistep clinical–radiological pathway failure, characterized by radiological undercharacterization, nonspecific report recommendations, and subsequent clinical anchoring bias on benign pathologies. Retrospective AI analysis successfully flagged the indeterminate renal lesion as suspicious, demonstrating its value as a supplementary decision‐support tool. However, the AI report’s critical factual errors—such as hallucinating contrast enhancement and mislocalizing the lesion—highlight that AI cannot replace the clinical oversight of experienced radiologists. Clinicians ordering imaging for flank pain must ensure adherence to standard diagnostic protocols, including contrast medium when indicated, and report recommendations must remain highly specific to avoid catastrophic diagnostic delays [13, 14].

## Funding

No funding was received for this study.

## Consent

Written informed consent for the publication of this comprehensive case report, including all clinical details, pathological findings, and accompanying anonymized imaging figures, was formally obtained from the patient’s next of kin (his adult children). The consenting parties reviewed the finalized manuscript text and figures and were fully informed of the potential risks of online re‐identification despite the complete removal of direct personal and clinical identifiers.

## Conflicts of Interest

The authors declare no conflicts of interest.

## Data Availability

Data sharing is not applicable to this article as no datasets were generated or analyzed during the current study.
